# MCM-2, Ki-67, and EGFR downregulated expression levels in advanced stage laryngeal squamous cell carcinoma

**DOI:** 10.1038/s41598-021-94077-9

**Published:** 2021-07-16

**Authors:** Sarocha Vivatvakin, Thanaporn Ratchataswan, Thiratest Leesutipornchai, Komkrit Ruangritchankul, Somboon Keelawat, Patnarin Mahattanasakul, Saknan Bongsebandhu-phubhakdi

**Affiliations:** 1 Department of Physiology, Faculty of Medicine, Chulalongkorn University, 1873 Rama 4 Road, Patumwan, Bangkok, 10330 Thailand; 2 Department of Pathology, Faculty of Medicine, Chulalongkorn University, Bangkok, Thailand; 3 Department of Otolaryngology, Head and Neck Surgery, Faculty of Medicine, Chulalongkorn University, Bangkok, Thailand; 4 Department of Otolaryngology, Head and Neck Surgery, King Chulalongkorn Memorial Hospital, Thai Red Cross Society, Bangkok, Thailand

**Keywords:** Diagnostic markers, Cancer screening, Cancer therapy, Head and neck cancer

## Abstract

We present the conceptual study investigated the capacity of minichromosome maintenance-2 (MCM-2), Ki-67, and epidermal growth factor receptor (EGFR) to assess the severity and progression of laryngeal squamous cell carcinoma (LSCC) disease and to study the correlations among these markers. A total of 30 patients with LSCC with immunohistochemistry (IHC) staining for MCM-2, Ki-67 and EGFR were examined. Mean expression levels of the three markers were evaluated for comparing between early and advanced stages of LSCC. The mean MCM-2, Ki-67, and EGFR expression levels were significantly decreased in advanced-stage compared with early-stage LSCC. Pearson correlation analysis showed a statistically significant correlation between the MCM-2 and Ki-67. Regarding subgroup analyses, MCM-2, Ki-67, and EGFR showed significant differences between early- and advanced-stage LSCC with non-recurrence, while for the recurrent subgroup LSCC, only MCM-2 revealed a significant difference between early- and advanced-stage LSCC. Altogether, these results support the role for downregulation of MCM-2, Ki-67 and EGFR in advanced-stage LSCC and correlation of MCM-2 and Ki-67 expressions that would be a promising strategy to predict prognosis of LSCC including severity and progression. We contextualize our findings and advocate the position of the biological markers, especially MCM-2, as an emerging evaluation tool for LSCC disease.

## Introduction

In this modern period of continuous medical development, several prognostic evaluations and treatment modalities for cancers have been invented according to the pathogenesis and pathophysiology of cancer, including laryngeal cancer^1^. Apart from tumor staging, other world-wide accepted molecular biomarkers for laryngeal squamous cell carcinoma (LSCC) have not yet been established. Well-known biological markers of cell proliferation, including MCM-2 and Ki-67, along with the proto-oncogene, EGFR, have been reported to play pivotal roles in the pathogenesis of several cancers, including LSCC^2,3^.

The current newcomer biomarker for LSCC prognostic prediction is the minichromosome maintenance (MCM) complex^4^. The MCM complex is generated at the beginning of the G1 phase and is fully activated in the S phase, where it unwinds the DNA through helicase activity^5^. After completing DNA replication, the MCM complex is exported from the cytoplasm and degraded to ensure that only one chromosome replication occurs each cell cycle^6^. Therefore, the detection of MCM proteins may be a key factor in tumor proliferation^7^. In addition, MCM proteins have been reported to have an impact on prognostic prediction in multiple neoplasms, especially MCM-2, which has outstanding potential as a biological marker of cell proliferation and prognostic markers for dysplasia, malignant transformation, and cancer^5,8^.

Another biomarker that has been widely used as a conventional marker for tumor proliferation in various cancers, including LSCC, is Ki-67^9^. Ki-67 can be detected in active cell cycle phases, including G1, S, G2, and M phases, with maximal expression in the G2 and early mitosis phases but less expression in the early G1 phase^10,11^. The level of Ki-67 then declines rapidly in anaphase and telophase during mitosis^11^. Additionally, its expression is rarely detected in quiescent cells or in cells during the G0 phase^11,12^. This variable expression pattern of Ki-67 allows it to be accepted as an indicator of cell proliferation in various cancers^13^. Currently, the only rational molecular-targeted therapy for LSCC is cetuximab (C225/Eribitux), a monoclonal antibody that inhibits the epidermal growth factor receptor (EGFR), which is expressed on tumor cells^3^. The roles of EGFR are not only in the regulation of normal cellular functions but also in the pathogenesis of several types of cancer, as EGFR is widely accepted a proto-oncogene^14^.

Discussion regarding the association between laryngeal cancer and the expression of these biological markers including MCM-2, Ki-67, and EGFR, has dominated research in recent years. Several studies have shown that the expression of both MCM-2 and Ki-67 is correlated with more severe clinical stages, pathological characteristics, lymph node metastasis and shorter survival in LSCC^4,9,15,16^. Moreover, studies on EGFR reported results in the same direction, indicating that elevated EGFR is associated with more invasive disease stages, aggressive histopathological features and poorer prognosis^17,18^. Nevertheless, conflicting outcomes in some previous studies were also noted. MCM-2 underexpression has been proposed to correlate with more tumour aggressiveness and worse prognosis in various types of malignancy^8,19^. Furthermore, decreased Ki-67 expression was documented to be correlated with radiotherapy unresponsiveness in head and neck squamous cell carcinoma (HNSCC)^20^. Contrasting information was also presented for EGFR, as a low level of EGFR expression was related to advanced stage disease, higher recurrence, and higher mortality rates in HNSCC, including LSCC^21^. Whereas, there was evidence that EGFR expression was not associated with the clinicopathological and prognostic indicators in LSCC^22^. The conclusions of these associations are still debatable. However, understanding the profiles of these delicately chosen biomarkers is essential. Alterations of these markers may crucially imply the degree of the malignancy in LSCC since they indeed play multiple important parts in the pathogenesis and pathophysiology of cancer.

The primary endpoint of the present study is defined as the capacities of MCM-2, Ki-67 and EGFR to serve as markers of LSCC disease severity characterized as early- and advanced-stage when LSCC patients were diagnosed and their possibilities to be prognostic markers of LSCC progression distinguished clinically between non-recurrence and recurrence. Secondary endpoint includes the correlations among these markers. Detection of these markers in patients with LSCC might be used as a novel tool for the early detection of disease progression to ensure timely, proper management of patients.

## Materials and methods

We performed all experiments and methods in accordance with relevant guidelines and regulations. All protocols in this study were approved by The Institutional Review Board of the Faculty of Medicine, Chulalongkorn University (IRB-MDCU) No. 562/58 (Bangkok, Thailand). Patient selection in this study was done according to the inclusion criteria that the patients were diagnosed with LSCC and presented at the King Chulalongkorn Memorial hospital (KCMH; tertiary care centre) between January 1, 2010, and December 31, 2014. There were 135 patients of LSCC retrieved from the files of Department of Pathology, KCMH. Of these, 43 were diagnosed with supraglottic carcinoma, six refused medical care, 15 were referred with pathological reports, eight were non-squamous cell carcinoma, 11 had lost medical records, one was referred to other hospital, two were recurrences before 2010 and 19 had incomplete follow up. Therefore, 30 patients were included in this study for further analysis. Follow up visits were totally scheduled in 8 years. There were 29 men and 1 woman with the age ranged from 43 to 80 years old. We obtained informed consent from all patients, and all methods were carried out in accordance with the relevant guidelines and regulations of IRB-MDCU. Patients included in this study received their treatment according to their disease stage; 12 were treated with radiotherapy (RT), two received concurrent chemoradiotherapy (CCRT), three underwent total laryngectomy (TLG), seven underwent TLG with postoperative RT and 6 underwent TLG with postoperative CCRT. Follow-up visits were totally scheduled in 8 years; every 4–8 weeks for the first 2 years, every 3 months for year 3, every six months for years 4 and 5 then once a year. The tissue specimens were collected before applying the chemotherapy and radiotherapy. Haematoxylin and eosin staining was performed for examination. The clinicopathological features are summarized in Table 1. All samples were staged according to AJCC TNM Cancer Staging Manual, 8th Edition^23^.

**Table 1:** Clinicopathological features of a total 30 LSCC patients.

Clinicopathological features	Total	MCM-2	p-value	Ki-67	p-value	EGFR	p-value
Total	30 (100%)	162.4333		32.9567		165.0090	
**Histological differentiation**			0.351		0.148		0.729
Well	17 (56.67%)	169.4471		40.4824		162.2394	
Moderate	13 (43.33%)	153.2615		23.1154		168.6308	
**Lymphatic metastasis**			0.478		0.499		0.135
LN negative	24 (80.00%)	161.6583		34.6375		173.9333	
Unilateral	3 (10.00%)	142.3333		40.3000		129.2233	
Bilateral	3 (10.00%)	188.7333		12.1667		129.4000	
**Metastasis**			0.614		0.574		0.579
No metastasis	25 (83.33%)	164.3840		31.4360		167.2748	
Metastasis	5 (16.67)	152.6800		40.5600		153.6800	
**Stage**			0.010^*^		0.078		0.001^*^
I	9 (30.00%)	198.2111		51.4778		184.3889	
II	1 (3.33%)	216.2000		73.5000		245.9000	
III	4 (13.33%)	150.0750		21.6000		212.3500	
IV	16 (53.33%)	142.0375		22.8437		137.2169	
**Recurrent**			0.416		0.523		0.128
No	15 (50.00%)	155.4200		29.0933		178.6713	
Yes	15 (50.00%)	169.4467		36.8200		151.3467	
**Survival**			0.495		0.538		0.087
> 5 years	17 (56.67%)	167.5824		36.2118		178.3747	
< 5 years	13 (43.33%)	155.7000		28.7000		147.5308	
Alive	16 (53.33%)	170.9000	0.219	36.6938	0.509	178.6294	0.103
Death	14 (46.67%)	152.7571		28.6857		149.4429	

^*^Differences were statistically significance.

### Immunohistochemistry

Formalin-fixed, paraffin-embedded blocks of laryngeal cancer tissue sections were cut at 3-μm thickness. Then the slides were stained in an automated immunostainer (Ventana, Benchmark XT) using primary antibodies of EGFR (CONFIRM anti-Epidermal Growth Factor Receptor (3C6) Primary antibody, Ventana, USA) and Ki-67 (CONFIRM anti-Ki-67 (30-9) Rabbit Monoclonal Primary Antibody, Ventana, USA) according to the manufacturer’s recommended protocol. For MCM-2 (anti-MCM2 rabbit monoclonal antibody (D7611)XP, Cell Signaling Technology, USA), the staining protocol (also using Ventana automated immunostainer, Benchmark XT) was modified by adding Standard CC1 as an antigen retrieval agent and incubating the specimens at 37 °C for 32 min. Distilled water was used as negative control replacing with primary antibodies. Brain, tonsil and colon adenocarcinoma were employed as positive controls of EGFR, Ki-67 and MCM-2, respectively.

### Scoring

Two pathologists evaluated all the stained slides. They were blinded to the clinical information. All MCM-2-, Ki-67-, and EGFR-stained slides were digitally imaged using the Aperio ScanScope XT (Aperio Technologies, Vista, CA). The immunostaining intensities were automatically scored using the Aperio ImageScope (v.12.1.0.5029) with commercially available Nuclear v.9 algorithm for scoring MCM-2 and Ki-67, and Membrane v.9 algorithm for scoring EGFR expressions. The semi-quantitative definition of grading category of nuclei or membranes is as follows; 0 (negative), 1+ (weak staining), 2+ (moderate) and 3+ (strong). Scores were given as numbers between 0 and 100 in each category^24^. The total expression scores of MCM-2, Ki-67 and EGFR were calculated according to the following formula.

$$\begin{aligned} {\text{Expression}}\;{\text{score}} & = \left( {\% \;{\text{of}}\;3 + {\text{nuclei}}/{\text{membranes}} \times 3} \right) + \left( {\% \;{\text{of}}\;2 + {\text{nuclei}}/{\text{membranes}} \times 2} \right) + \left( \% \right. \\ & \quad \left. {{\text{of}}\;1 + {\text{nuclei}}/{\text{membranes}} \times 1} \right) \\ \end{aligned}$$

### Statistical analysis

Data analysis was calculated by SPSS Statistics, Version 23.0. (Armonk, NY, USA.). In the evaluation of the data, mean ± standard deviation, and number (percentages) of patients were reported. The candidate independent variables included age, gender, clinicopathological characteristics of the patients, including histological differentiation, LN metastasis, disease metastasis, disease stage, tumour recurrence, and 5-year survival. In categorical comparisons, Pearson’s chi-square and Fisher’s exact tests were used to analyze the correlation between categorical variables. In continuous comparison, independent Student’s t-test and analysis of variance (ANOVA) was used. The correlation of each marker was calculated using Pearson’s correlation. The significance of all the tests was evaluated at the level of 95% (p < 0.05).

## Results

A total of 30 patients with primary laryngeal cancer were included in this study. All were eligible for analysis. There were 29 (96.67%) males and 1 (3.33%) female. The average age in this study was 66.04 years, with maximal and minimal ages of 80 and 43 years, respectively. The demographic data and clinicopathological characteristics of the patients, including histological differentiation, LN metastasis, disease metastasis, disease stage, tumour recurrence, and 5-year survival, are shown in Table 1. No differences in the expression of these biomarkers in the mentioned characteristic features were observed except for disease staging, which revealed a statistical relationship between mean MCM-2 and EGFR.

**Figure 1:**
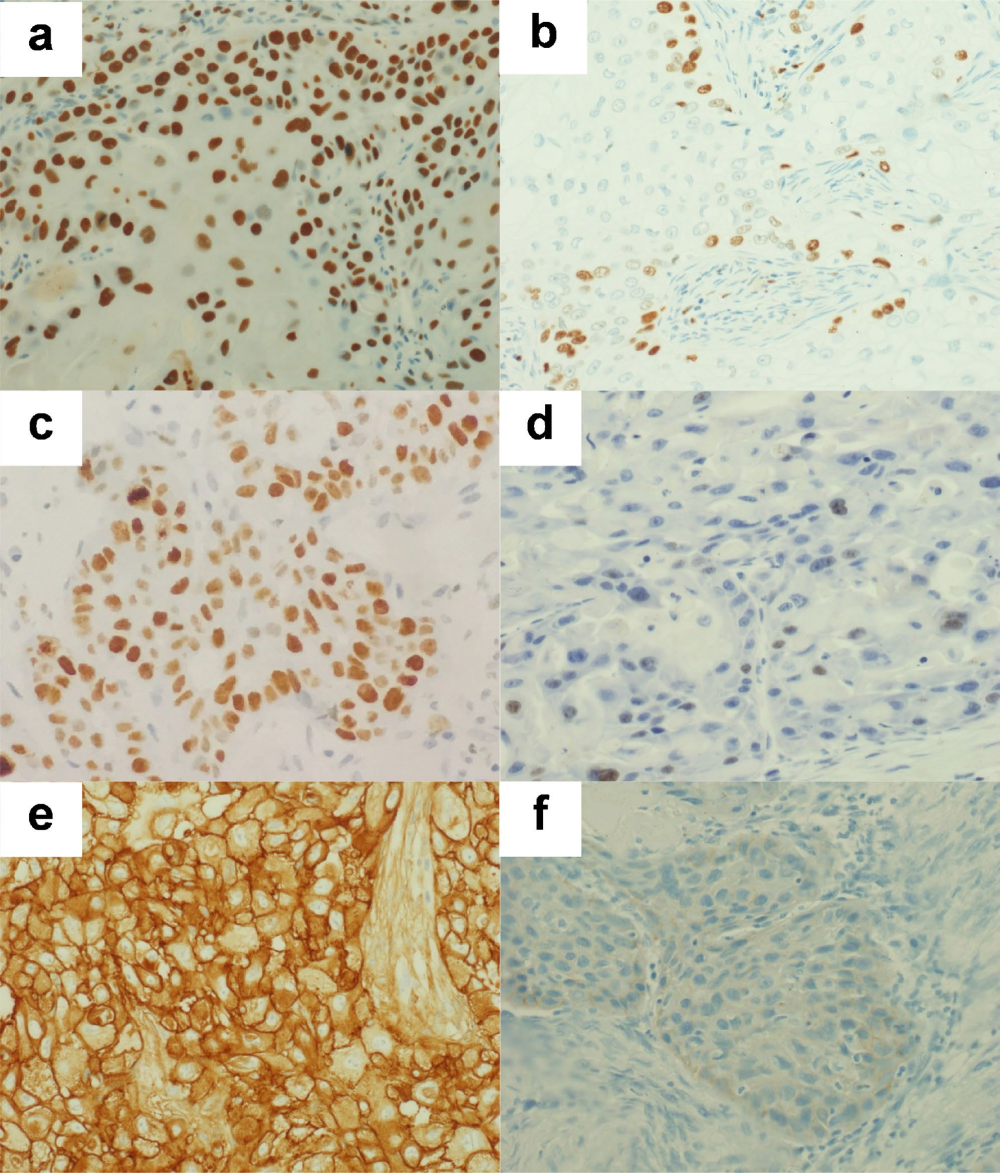
MCM-2, Ki-67 and EGFR immunoreactivity in representative laryngeal samples. Immunodetection performed using nuclear staining for MCM-2 (**a**, **b**) and Ki-67 (**c**, **d**), and membranous staining for EGFR (**e**, **f**) labeling in laryngeal squamous cell carcinoma. There are strong reaction in early stage (**a**, **c**, **e**), while MCM-2, Ki-67 and EGFR expressions are downregulated in advanced stage (**b**, **d**, **f**).

**Figure 2:**
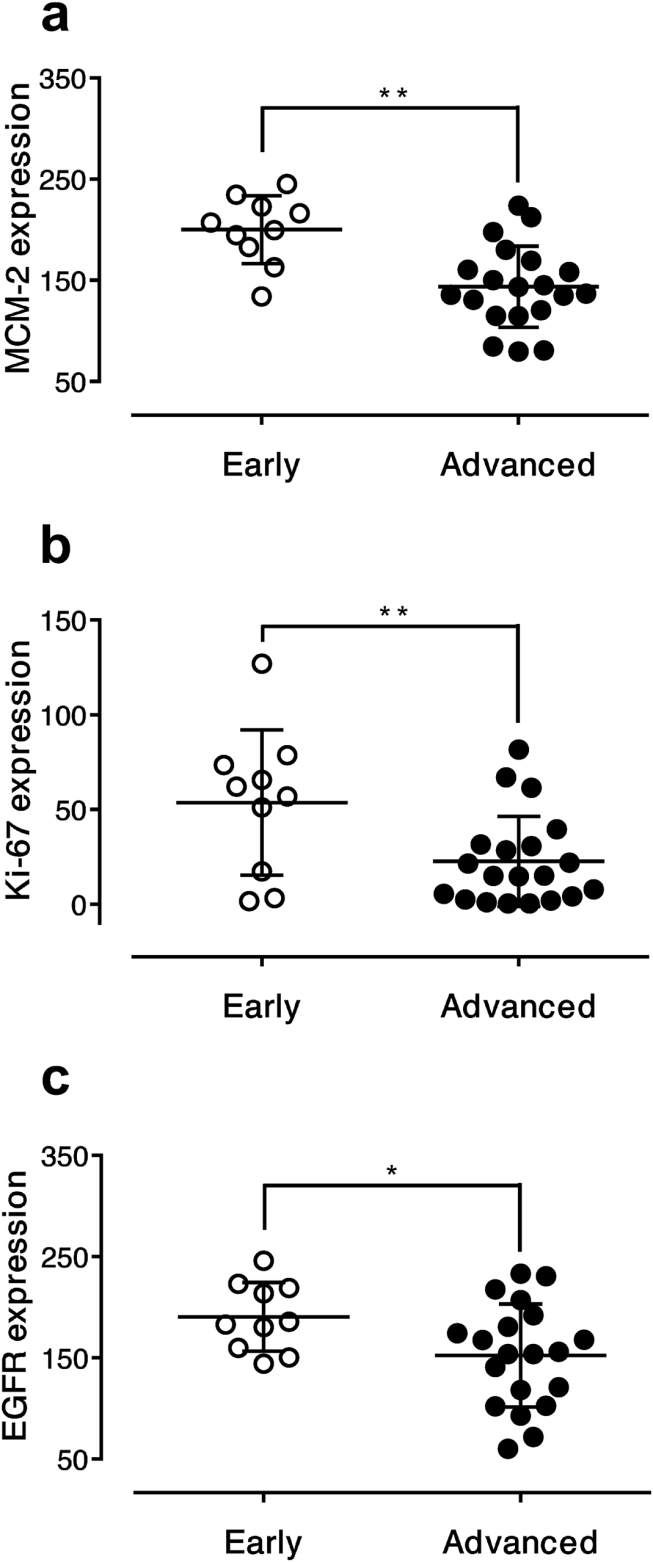
Significant downregulation of the mean expression levels of (**a**) MCM-2, (**b**) Ki-67, and (**c**) EGFR in advanced-stage disease in 30 LSCC patients. Data are presented as the mean ± SD. *p < 0.05, **p < 0.01.

The early stage of LSCC was defined as patients with stage 1 and 2, while the advanced stage was defined as patients with stage 3 and 4 LSCC. After immunostaining for MCM-2, Ki-67 and EGFR, the majority of LSCC showed some degree of positive staining and there was considerable downregulation of these biomarkers in comparison between early and advanced stage (Fig. 1). The mean expression levels of all three markers were calculated for the early and advanced stages of disease. Ten patients were classified as early stage, while twenty patients were classified as advanced stage. The mean MCM-2 expression levels were 200.01 ± 33.49 and 143.65 ± 40.20 for early and advanced stages, respectively (56.37; *p* = 0.001). The mean Ki-67 expression levels were 53.68 ± 38.28 and 22.60 ± 23.78 for early and advanced stages, respectively (31.09; *p* = 0.008). The mean EGFR expression levels were 190.54 ± 33.95 and 152.24 ± 50.82 for early and advanced stages, respectively (38.30; *p* = 0.041). The expression levels of all these markers in advanced-stage disease were significantly downregulated compared with early-stage disease (Fig. 2).

**Figure 3:**
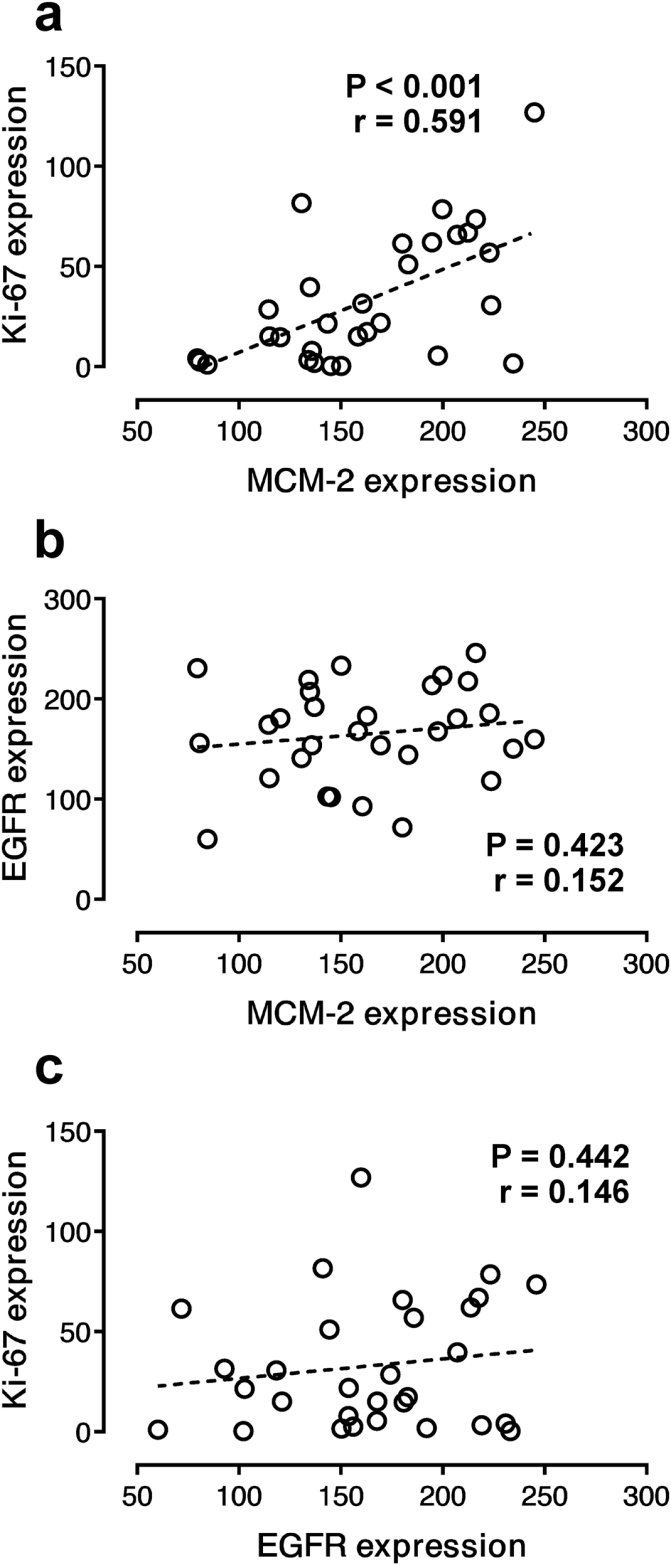
Pearson’s correlation between the expression levels of MCM-2, Ki-67, and EGFR in LSCC. (**a**) The correlation between MCM-2 and Ki-67 (*r* = 0.591; *p* < 0.001). (**b**) The correlation between MCM-2 and EGFR (*r* = 0.152; *p* = 0.423). (**c**) The correlation between Ki-67 and EGFR (*r* = 0.146; *p* = 0.442).

The correlations of these markers were investigated in this study (Fig. 3). Pearson correlation analysis showed a statistically significant relationship between the expression levels of the two proliferative markers, MCM-2 and Ki-67. A strong positive correlation between the expression levels of these two markers was revealed (*r* = 0.591; *p* < 0.001). However, the correlations between MCM-2 and EGFR expression and Ki-67 and EGFR expression showed no significant relationship (*r* = 0.152; *p* = 0.423 and *r* = 0.146; *p* = 0.442, respectively).

The mean follow-up time was 3.92 ± 2.46 years (range, 0.79 – 8.01 years), and the median follow-up time was 3.43 years. During the follow-up time of 8 years, recurrence was observed in 15 patients (50.00%). A total of ten cases (66.67%) recurred at the local site; three cases (20.00%) revealed distant metastases at the skin, lung, chest wall and esophagus, and two cases (13.33%) recurred at both local and distant organs (lung and liver). This study further investigated the tendencies of the expression levels of these three markers regarding recurrence. Half of the patients were classified as non-recurrence, while the other half was classified as recurrence. In the non-recurrence group, the mean expression levels of MCM-2, Ki-67 and EGFR were calculated for the early and advanced stages. The mean expression levels of these markers were also calculated similarly for the early and advanced stages in the recurrence group.

**Figure 4:**
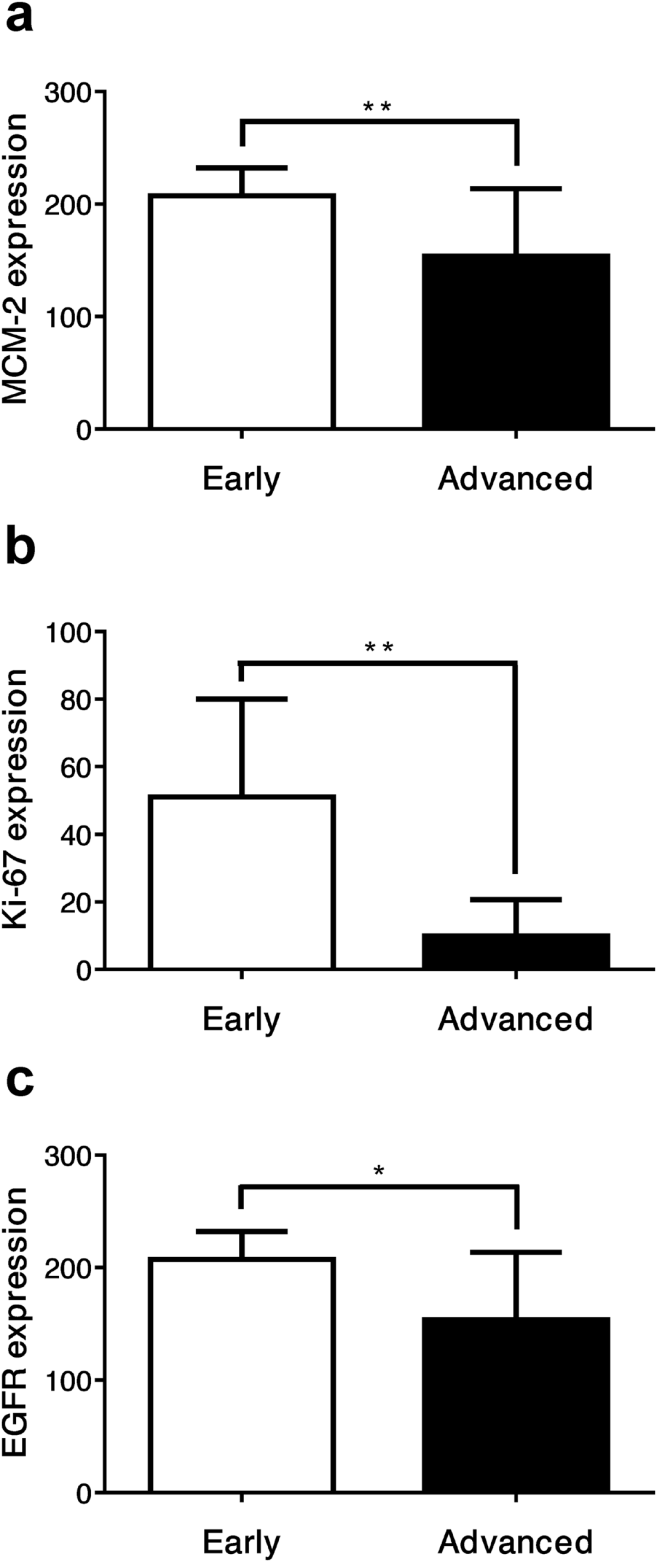
Significant downregulation of the mean expression levels of (**a**) MCM-2, (**b**) Ki-67, and (**c**) EGFR in advanced-stage disease of the 15 LSCC patients without recurrence. Data are presented as the mean ± SD. *p < 0.05, **p < 0.01.

Regarding the non-recurrence group, the mean MCM-2 expression levels were 191.07 ± 31.68 and 124.23 ± 31.68 for early and advanced stages, respectively (66.85; *p* = 0.001). The mean Ki-67 expression levels were 51.03 ± 29.04 and 9.90 ± 10.79 for early and advanced stages, respectively (41.13; *p* = 0.009). The mean EGFR expression levels were 207.27 ± 24.89 and 153.65 ± 59.94 for early and advanced stages, respectively (53.63; *p* = 0.047). The expression levels of all these markers in the advanced-stage non-recurrence group also showed significantly downregulated patterns compared with early-stage disease (Fig. 4).

**Figure 5:**
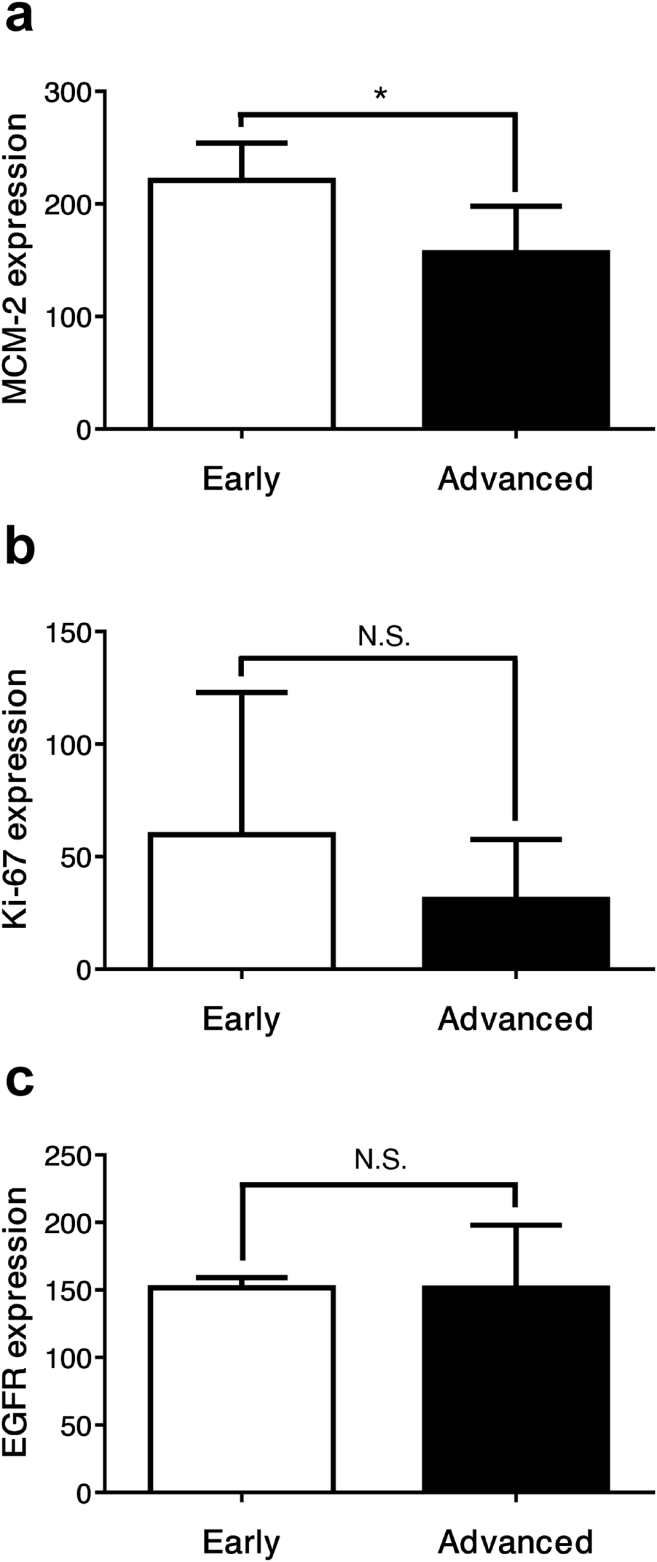
Significant downregulation of the mean expression levels of MCM-2 in the advanced-stage disease of the 15 LSCC patients with recurrence (**a**). Decreased mean expression levels of (**b**) Ki-67 and (**c**) EGFR in the advanced-stage disease of the 15 LSCC patients with recurrence. Data are presented as the mean ± SD. *p < 0.05, N.S. = non-significance, *p* > 0.05.

In the recurrence group, the mean MCM-2 expression levels were 220.87 ± 33.20 and 156.59 ± 41.18 for early and advanced stages, respectively (64.28; *p* = 0.027). The mean Ki-67 expression levels were 59.87 ± 63.11 and 31.06 ± 26.59 for early and advanced stages, respectively (28.81; *p* = 0.222). The mean EGFR expression levels were 151.50 ± 7.87 and 151.31 ± 46.61 for early and advanced stages, respectively (0.19; *p* = 0.989). The MCM-2 expression was the only marker that showed a statistically significant downregulation. Even though the other markers revealed insignificant differences, the expression levels of both Ki-67 and EGFR in the advanced stage of the recurrence group were still lower than in the early stage (Fig. 5). At the end of this study, 14 patients (46.67%) died, of which seven patients (50.00%) died from laryngeal cancer; one patient (7.14%) died from other causes, including myocardial infarction; and six patients (42.86%) died from unknown etiology.

## Discussion

This study was conducted to investigate the correlations of these three markers and the disease progression of patients with LSCC in terms of disease severity evaluation and cancer recurrence rate. The demographic data regarding to male-to-female ratio and age incidence are consistent with epidemiological information of the previous study^25^. The results showed dramatic downregulation of the expression levels of all the markers in more advanced-stage disease, especially when the disease was classified as the advanced stage. Only MCM-2 and Ki-67 showed a significant correlation. In addition, the results revealed a significantly lower MCM-2 level in the advanced stage of cancer in both recurrent and non-recurrent subgroup analyses. Ki-67 and EGFR demonstrated tendencies for lower expression in advanced-stage disease without statistical significance when subgroup analyses were applied. Even though the results represented counter-intuitive outcomes, several recent studies showed results that were consistent with the present study.

Increased MCM-2 expression was related to longer overall survival in patients with colorectal cancer^19^. Furthermore, non-small cell lung cancer (NSCLC) patients with lower MCM-7 expression tended to have concomitant bronchioloalveolar carcinomas compared with those with higher MCM-7 expression^6^. Although the relationship between a higher proliferation rate and a better prognosis may seem contrary to normal instinct, systematic explanations of this phenomenon have been documented. First, the higher proliferative rate of the tumors may be accompanied by a higher apoptotic rate of the tumor cells^19^. This explanation was related to a better prognosis in colorectal cancer with higher MCM-2 expression^19^. Second, the decrease in the expression of the MCM complex may influence its capacity to surveil DNA replication, resulting in chromosomal abnormality^26^. According to Das’s review of the MCM complex, this complex consists of the active MCM form, which functions as an initiator for DNA replication, and the inactive MCM form, which is the form that is detected in most cases^26^. Even though it is inactive for DNA replication, this form of MCM has been documented to play essential roles in replication repair and erratic cell proliferation defense^27^. The depletion of this inactive MCM will lead to hypersensitivity to replication stress, chromosomal aberration, genomic instability and eventually cancer development^28^. Third, the rapid overgrowth of aggressive tumors may lead to an inadequate blood supply for the tumor as the growth rate outweighs the angiogenesis, resulting in a hypoxic microenvironment^29^. The hypoxic condition suppresses the ubiquitination and proteasomal degradation of the hypoxia-inducible factor (HIF); thus, HIF expression increases and functions in cellular adaptation to the low-oxygen environment^30^. From Hubbi’s study in human embryonic cell culture, MCM is downregulated in hypoxic conditions with the presence of HIF; however, its downregulation was not observed when HIF was knocked down^30^. Fourth, even though the MCM complex consists of six protein subunits, including MCM-2, 3, 4, 5, 6, and 7, the helicase activity is mainly mediated by MCM-4, -6 and -7, while other proteins have other roles during replication^31,32^. These three proteins form a stable trimeric complex, and MCM-2 is loosely attached to this complex^32^. From Ishimi’s study, helicase activity can be detected only in the MCM complex extracted from HeLa cells with MCM-2 disappearance^32^. Therefore, MCM-2 expression may prevent MCM-4, -6 and -7, assembly resulting in DNA replication inhibition. The specificity of MCM-2 may be the reason for the significant difference in MCM-2 expression between early and advanced stages of LSCC in both the recurrent and non-recurrent subgroups.

Regarding Ki-67, Ki-67 expression was found to be directly related to LSCC recurrence after treatment^33^. However, it was also shown that elevated Ki-67 levels were associated with better local control and more radiotherapy sensitivity in head and neck cancer, as higher proliferative neoplasms might be more responsive to radiation treatment and result in better prognosis^20^. There is evidence that Ki-67 vanishes as laryngeal squamous cancer cells are transformed into squamous pearls, which may explain the downregulation of Ki-67^18^. Ki-67 plays a role not only in tumour proliferation but also in the stabilization of DNA replication^34,35^. During mitosis, Ki-67 is the main protein component of the perichromosomal layer (PCL), which persists throughout mitosis, preventing chromosome aggregation^34,35^. The diminishment of Ki-67 interferes with the PCL formation, which may result in asymmetrical distribution of daughter cells, disruption of spindle assembly, prolongation of cell division progression from prometaphase to anaphase, impairment of mitotic chromosome structures and malignancy progression^35^.

Attenuation of EGFR expression was found in high-grade tumor cells in various types of HNSCC^36,37^. Furthermore, patients with membranous EGFR immunoreactivity of more than 10% had longer survival rates with OSCC and nasal sinus SCC compared to those with less EGFR expression^14,21^. Various hypotheses have been proposed to explain the downregulation of EGFR in high-grade malignant cells. One of the mechanisms is hypoxia, which has similar pathophysiological effects as MCM. Hypoxia enhances the upregulation of prolyl hydroxylase 3 (PHD3), one of the HIFs, which in turn causes EGFR internalization via endocytosis, leading to the downregulation of EGFR^29^. Not only does hypoxia play an important role in EGFR suppression, but other physiologic mechanisms are also involved in this mechanism, such as nutritional shortage and local metabolic waste accumulation^38^. Another explanation is that some high-grade invasive OSCC cells have an elevated level of epithelial-mesenchymal transition, which then leads to EGFR suppression^36^. This may be the reason why OSCC with overexpression of EMT-associated genes seems to be cetuximab-resistant^36^.

This study also demonstrated that MCM-2 was strongly correlated with Ki-67 expression, while others showed no significant relationship. Several previous studies have also advocated a similar correlation between MCM proteins and Ki-67 in HNSCC^17,39,40^. Ki-67 has been widely used as a conventional marker for cell proliferation; thus, MCM-2 may have the same potential as Ki-67 for cancer progression evaluation^8,41,42^. Moreover, this and other recent studies have shown that the MCM proteins might be an alternative tool that is superior to or even be able to replace Ki-67^8,41,42^. In this study, MCM was the only marker that could distinguish between early and advanced stages of disease in both the non-recurrent and recurrent groups. Furthermore, there are multiple reasons that MCM protein is suggested to be more favorable and practical than Ki-67. Primarily, MCM expresses more than Ki-67, as detected MCM includes both the active and the inactive or dormant forms^22^. Thus, MCM protein has better sensitivity compared with Ki-67^3,8,17,41^. Additionally, MCM-2 protein is a capable marker of cell proliferation^27^. MCM complex outstandingly presents throughout the whole cell cycle, including cells in the G0 phase that are preparing to begin the G1 phase^27^. Ki-67 is presented in a shorter duration, mainly the G2 and S phases^8^. As a result, Ki-67 is unable to identify cells with proliferative potential, which is still in the G0 phase^6,27^. Therefore, MCM protein has greater specificity for the evaluation of precancerous and cancerous tissue^3,8^.

The preliminary evidence from this study may be potentially relevant for practical guidance for implementing predictive biomarkers into prognostic importance of LSCC disease. Monitoring these markers levels in patients with LSCC might be applied as a practical tool for the early detection of disease progression to offer appropriate therapy to patients. For example, MCM-2 might be a novel beneficial biomarker for LSCC progression prediction. Moreover, a combination of multiple markers may provide additional information that is essential for disease progression assessment^43^. EGFR might also be used along with MCM-2 and Ki-67 for malignancy evaluation, even though a definitive correlation was not shown. EGFR is the only protooncogene in this study that may not correlate with the other two proliferative biomarkers^3^. Nevertheless, these proliferative markers may still be able to function as an independent prognostic tool^44^.

Some potential limitations in this framework should be addressed. This study was confined only to the King Chulalongkorn Memorial Hospital, with a quite small sample size. Despite the sample size, the data observed in this study still showed statistical significance. The findings from this study confirm that the use of immunohistochemical staining is sufficient to quantify the expression scores of MCM-2, Ki-67 and EGFR, as same as in many previous studies^45–47^. Further, this study was followed over 8 years which is relatively appropriate follow-up time for LSCC. Additionally, assessments of the expression levels of MCM-2, Ki-67 and EGFR using the scoring system in this study were performed without interpathologist variation bias.

Further study with a larger number of subjects should be developed to strengthen the results of this study. Alterations of these biomarkers may represent additional significant results of other parameters. In the MCM complex, MCM proteins other than MCM-2 should also be investigated for possible distinct findings. Moreover, labelling detecting the active and inactive forms of MCM protein may help improve the knowledge of the MCM mechanisms that are involved in the pathophysiology of cancer development and tumor progression.

## Conclusions

The downregulation of the expression levels of biological markers, including MCM-2, Ki-67, and EGFR, is correlated with more advanced stage LSCC. This study reinforces the positions of the biological markers, especially MCM-2, as an evaluation tool for LSCC disease progression. In conformity with these findings, assessments of MCM-2, Ki-67, and EGFR in LSCC patients should be considered, as the benefit of early detection of disease severity is highly valuable.
